# COVID-19 Vaccine Hesitancy Among Pregnant and Lactating Mothers Attending Government Health Care Centers in Karaikal, South India

**DOI:** 10.7759/cureus.41456

**Published:** 2023-07-06

**Authors:** Niranjjan R, S Nancy, Arulvijayavani S, Nanthini Saravanan, S Sathish Kumar

**Affiliations:** 1 Department of Community Medicine, Vinayaka Mission’s Medical College and Hospital, Vinayaka Mission’s Research Foundation, Karaikal, IND; 2 Department of Biochemistry, Jawaharlal Institute of Postgraduate Medical Education and Research, Karaikal, IND; 3 Department of Obstetrics and Gynaecology, Jawaharlal Institute of Postgraduate Medical Education and Research, Karaikal, IND

**Keywords:** primary care, lactating mothers, pregnant mothers, vaccine hesitancy, covid-19

## Abstract

Introduction and aim: The Government of India has endorsed COVID-19 vaccination for pregnant and lactating mothers. However, the vaccine acceptance rate among this target group is not satisfactory. The study aimed to assess the awareness level, acceptance rate, and hesitancy for COVID-19 vaccination among pregnant and lactating mothers attending government health care centers and to identify the psycho-social factors associated with vaccine hesitancy.

Materials and methods: A government facility-based cross-sectional study was conducted in various government primary and secondary health care centers in Karaikal for a period of six months. After Institutional Ethics Committee (IEC) clearance, a semi-structured questionnaire was used to collect the data from 904 pregnant and lactating mothers. Bivariate and multivariate logistic regression were employed.

Results: Despite a high awareness level (87%), vaccine hesitancy was high (55%) among the study participants. In multivariate analysis, age > 30 years, primi mothers, lactating mothers with < 6 months old children, unawareness, and recent COVID-19 infection were significantly associated with vaccine hesitancy. Fear of side effects for baby and mother following vaccination and family pressure were the prime reasons for vaccine hesitancy.

Conclusion: Despite sufficient awareness about the eligibility for COVID-19 vaccination, the acceptance rate was low. There is a dire need to motivate the higher age group, primigravidas, and lactating mothers at the community level to get rid of their fear factors related to vaccination.

## Introduction

Since the first case of coronavirus disease (COVID-19) was reported in 2019, there have been more than 440 million people infected globally, leading to more than 5.9 million deaths up to early March 2022. After the rapid development and licensing of COVID-19 vaccines in 2021, 10.88 billion doses have been administered globally. About 63.3% of the population around the world received at least one dose of the COVID-19 vaccine [1]. Even though vaccination in pregnancy prevents maternal and infant morbidity and mortality, pregnant women have varied concerns regarding vaccine uptake [2,3]. Emerging evidence suggests that pregnant mothers with COVID-19 infection are at higher risk of maternal complications, intensive care unit admission, and the need for invasive ventilation when compared with non-pregnant patients of the same age. Elderly primi, pregnant women with high body mass index, diabetes, and hypertension had a poor prognosis with COVID-19 infection. Therefore, pregnant women are classified as a high-risk population for COVID-19 infection [2,3].

The government of India provided approval for the vaccination of pregnant women against COVID-19 on July 2, 2021. Lactating women were recommended to receive COVID-19 vaccinations in May 2021 [4]. The benefits of vaccinating pregnant and lactating women seem to far outweigh the theoretical and remote risks of undergoing vaccination. Lactating women should also be included as COVID vaccine candidates, as there are no known adverse effects on breastfeeding infants. There is a possibility of transplacental transmission of protective antibodies to the fetus, which may have a beneficial effect [5,6].

The success of a vaccine is determined not only by its efficacy but also by its acceptance rate. However, World Health Organization (WHO) pointed out that vaccine hesitancy has become a huge threat to public health. Several important key factors responsible for vaccine hesitancy include fear or mistrust of the vaccine, underestimation of the value of the vaccine, and lack of access to the vaccine [7]. Further, the evidence regarding the safety of COVID-19 vaccines during pregnancy and breastfeeding is limited. This has raised concerns among pregnant and lactating women and healthcare providers, with some casting doubts, contributing to vaccine hesitancy [7]. Also, the exclusion of pregnant and lactating women from clinical trials contributes to the lack of data. Inconsistencies in recommendations on COVID-19 vaccination published by expert advisory groups may contribute to patients delaying or refusing to accept vaccination [3].

Despite tremendous improvements in vaccine development and administration, the current acceptance level of the COVID-19 vaccine remains inadequate to meet the requirements for achieving herd immunity. The herd immunity threshold depends on the basic reproduction number of the disease. Assuming a basic reproductive number of 4, the community immunity level needs to reach at least 75% to stop the COVID-19 pandemic [8]. WHO has categorized vaccine hesitancy as one of the top ten threats to global health, even before the COVID-19 pandemic. Therefore, it is crucial to understand and address the reason for vaccine hesitancy [8]. Consequently, the present study was planned to assess the awareness level, acceptance rate, and hesitancy for COVID-19 vaccination among pregnant and lactating mothers attending government healthcare centers and to identify the psycho-social factors associated with vaccine hesitancy.

## Materials and methods

Study setting and sampling

A government facility-based cross-sectional study was conducted in various government healthcare facilities (primary health centers, community health centers, and government hospitals) in Karaikal. Those health centers situated within five kilometers of our tertiary care hospital were included in this study. The sample size is 904 (calculated using Open_Epi 3.01 software). All pregnant and lactating women attending these health centers during the study period (October 2021 to March 2022) were included in this study consecutively. Antenatal, postnatal, and immunization clinics were utilized to interview pregnant and lactating women, respectively. Inclusion criteria were (i) any women with confirmed intrauterine gestation, regardless of their gestational age, attending antenatal clinics; (ii) any women currently breastfeeding their babies attending immunization or postnatal clinics; or (iii) having a child less than one year of age, irrespective of breastfeeding status. Women younger than 18 were excluded from the study. These special clinics were scheduled every Wednesday and Thursday.

Data collection

Before conducting this study, permission from the Deputy Director of Immunization and Institute Ethical Committee approval (IEC approval no.: VMMC/CM/2021/78) was obtained. With written informed consent, face-to-face interviews were carried out with strict COVID-19 precautionary measures. A pre-tested semi-structured questionnaire was used to collect information like socio-demographic profile, order of pregnancy, number of children, trimester, breastfeeding week/month, place of care, morbidity details, history of COVID-19 infection, awareness about COVID-19 vaccination, vaccination status, number of doses, adverse effects following immunization, reasons for hesitancy and acceptance of vaccination. Adverse events following immunization such as pain, redness, swelling, tiredness, headache, myalgia, fever, chills, and nausea (mild events) and anaphylaxis, thrombosis with thrombocytopenia syndrome, Guillain-Barre Syndrome, myocarditis and pericarditis were assessed (CDC). The questionnaire was pre-tested with 10% of the sample to improve its validity and reliability. The primary study outcome was COVID-19 vaccine hesitancy, which was defined in accordance with the World Health Organization (WHO) Strategic Advisory Group of Experts (SAGE) on vaccine hesitancy as “uncertainty or refusal of vaccination, despite the availability of vaccination services [8]."

Data analysis

Data were collected using Epicollect5 Android software, converted into MS Excel (Redmond, USA), and analyzed using IBM Corp. Released 2013. IBM SPSS Statistics for Windows, Version 22.0. Armonk, NY: IBM Corp. Bivariate and multivariate analyses were used to determine the factors associated with vaccine hesitancy among pregnant and lactating mothers. Significant variables in the bivariate analysis are taken for multivariate analysis (logistic regression). The multiple coefficients of determination (R2) were employed as the goodness-of-fit statistic for the model, and statistical significance was fixed at 5% (P < 0.05).

## Results

Out of 904 study participants, 54.1% were pregnant women, and 45.9% were lactating women. The majority of the mothers, 53.7%, belonged to the age group of 24 to 29 years, and almost 86.5% practiced Hinduism. The mean age of the study participants was 26.6 ± 3.9 (SD) years. More than 90% of the study participants received above middle school education, and only 10% of the study participants were employed. Nearly 66.2% belonged to higher socio-economic classes (Table 1).

**Table 1:** Socio-demographic and other factors associated with vaccine hesitancy among pregnant and lactating mothers (N=904)

Variables	N (%) 904 (100%)	Received vaccine 408 (45%)	Not received vaccine 496 (55%)	OR (95% CI)	aOR (95%CI)
Religion					
Hindu	782 (86.5)	357 (45.7)	425 (54.3)	1	1
Muslim	59 (6.5)	26 (44.1)	33 (55.9)	1.0 (0.6 – 1.8)	0.7 (0.4 – 1.3)
Christian	63 (7.0)	25 (39.7)	38 (60.3)	1.2 (0.7 – 2.1)	0.7 (0.3 – 1.6)
Age group					
18 - 23	238 (26.3)	110 (46.2)	128 (53.8)	1	1
24 - 29	485 (53.7)	219 (45.2)	266 (54.8)	1.0 (0.7 – 1.4)	1.1 (0.8 – 1.6)
>= 30	181 (20.0)	79 (43.6)	102 (56.4)	1.1 (0.7 – 1.6)	1.6 (1.0 – 2.6)
Education					
Below Middle School	76 (8.4)	29 (38.2)	47 (61.8)	1.3 (0.8 – 2.2)	0.9 (0.5 – 1.6)
Above Middle School	828 (91.6)	379 (45.8)	449 (54.2)	1	1
Occupation					
Unemployed	814 (90.0)	366 (45.0)	448 (55.0)	1.0 (0.6 – 1.6)	1.1 (0.6 – 1.7)
Employed	90 (10.0)	42 (46.7)	48 (53.3)	1	1
Socio-economic Status (Modified BG Prasad Classification)					
Class 1 & 2	598 (66.2)	278 (46.5)	320 (53.5)	1	1
Class 3 & above	306 (34.8)	130 (42.5)	176 (57.5)	1.1 (0.8 – 1.5)	1.2 (0.9 – 1.6)
Trimester (n = 489)					
1 (Conception to > 12 weeks)	104 (21.2)	56 (53.8)	48 (46.2)	1	
2 (12 weeks to > 24 weeks)	201 (41.2)	92 (45.7)	109 (54.3)	1.3 (0.8 – 2.2)	
3 (24 weeks to 40 weeks)	184 (37.6)	81 (44.0)	103 (56.0)	1.4 (0.9 – 2.4)	
Order of pregnancy					
Primi	564 (62.4)	229 (40.6)	335 (59.4)	1.6 (1.2 – 2.1)	1.7 (1.2 – 2.4)
2^nd^ and above	340 (37.6)	179 (52.6)	161 (47.4)	1	1
Number of children					
Zero	298 (33.0)	122 (40.9)	176 (59.1)	1.2 (0.9 – 1.6)	1.1 (0.6 – 1.9)
One and above	606 (67.0)	286 (47.2)	320 (52.8)	1.4 (0.9 – 2.0)	1.0 (0.4 – 2.5)
Breastfeeding months (n = 415)					
< = 6 months	272 (65.5)	94 (34.6)	178 (65.4)	2.7 (1.8 – 4.2)	2.9 (1.8 – 4.6)
>6 months	143 (34.5)	85 (59.4)	58 (40.6)	1	1
At least one morbidity					
No	777 (86.0)	357 (46.0)	420 (54.0)	1	1
Yes	127 (14.0)	51 (40.2)	76 (59.8)	1.2 (0.8 – 1.8)	1.2 (0.8 – 1.9)
Awareness about vaccine					
No	113 (12.5)	16 (14.2)	97 (85.8)	5.9 (3.4 – 10.2)	6.6 (3.3 – 11.7)
Yes	791 (87.5)	392 (49.6)	399 (50.4)	1	1
COVID-19 infection					
No	842 (93.1)	392 (46.6)	450 (53.4)	1	1
Yes	62 (6.9)	16 (25.8)	46 (74.2)	2.5 (1.3 – 4.4)	2.4 (1.3 – 4.4)
Subject					
Pregnant women	489 (54.1)	229 (46.8)	260 (53.2)	1	1
Lactating women	415 (45.9)	179 (43.1)	236 (56.9)	1.1 (0.8 – 1.5)	1.2 (0.4 – 3.7)

Among 489 pregnant mothers, 41.2% were in the second trimester of the antenatal period, and among 415 lactating mothers, 65.5% were breastfeeding their child within six months. The majority, 62.4% of mothers, were primigravidas. About 67% had one or more living children in the family. Notably, 87.5% had adequate knowledge about the COVID-19 vaccine, and 6.9% had a recent COVID-19 infection (Table 1).

Notably, almost 55% of pregnant and lactating mothers attending government health facilities in Karaikal didn’t receive the COVID-19 vaccine. In bivariate analysis, primi mothers breastfeeding less than six months and having no awareness or history of COVID-19 infection were associated with vaccine hesitancy. Further, in multivariate analysis, ages more than 30 years, primi mothers, lactating mothers with less than six-month-old children, and women with no awareness or history of COVID-19 infection were significantly associated with vaccine hesitancy (Table 1).

**Figure 1:**
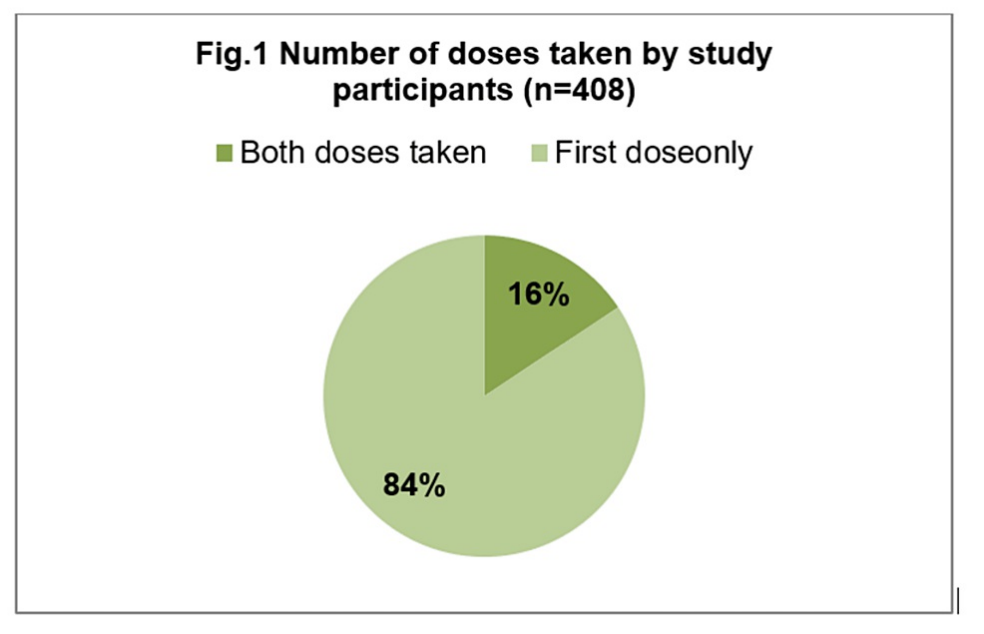
Number of doses taken by study participants [n = 408]

**Figure 2:**
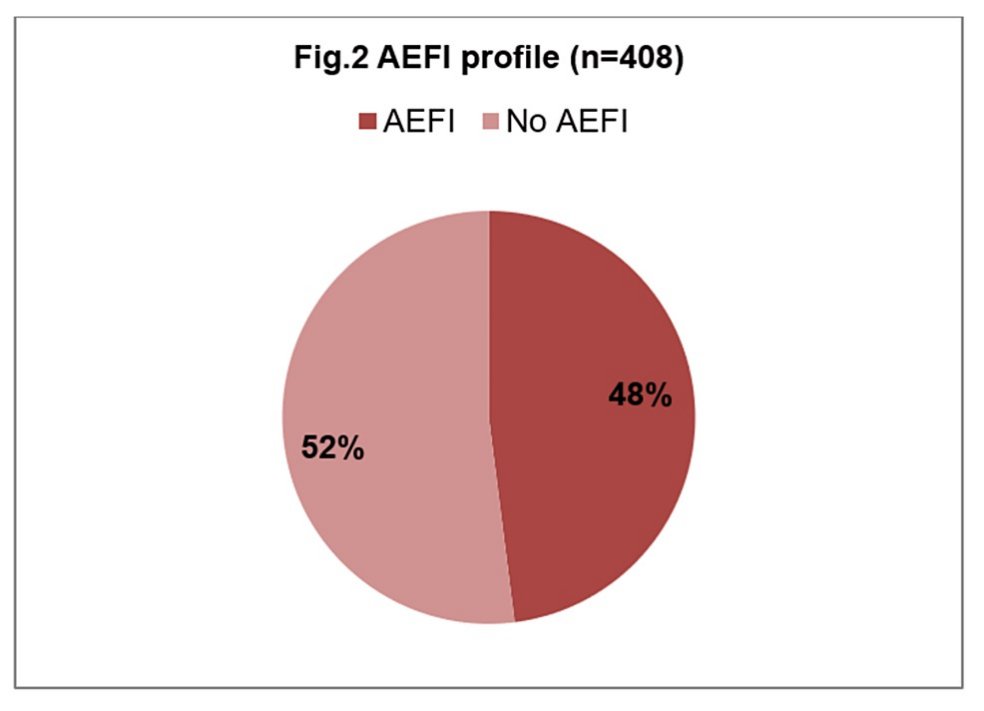
AEFI Profile [n = 408]

Among the 408 (45%) mothers who received COVID-19 vaccination, 345 (84.5%) had taken the first dose of vaccine, and only 63 (15.4%) had taken two doses of vaccine (Figure 1). Among those vaccinated, only 16% of them completed both doses of vaccine, and for the remaining 84% of those who received their first dose, a majority reported that the time limit for the second dose had not yet been reached. Nearly half of the vaccinated mothers experienced Adverse Effects Following Immunization (AEFI) (Figure 2). The most common adverse effects were fever, followed by body aches and injection site pain.

**Figure 3:**
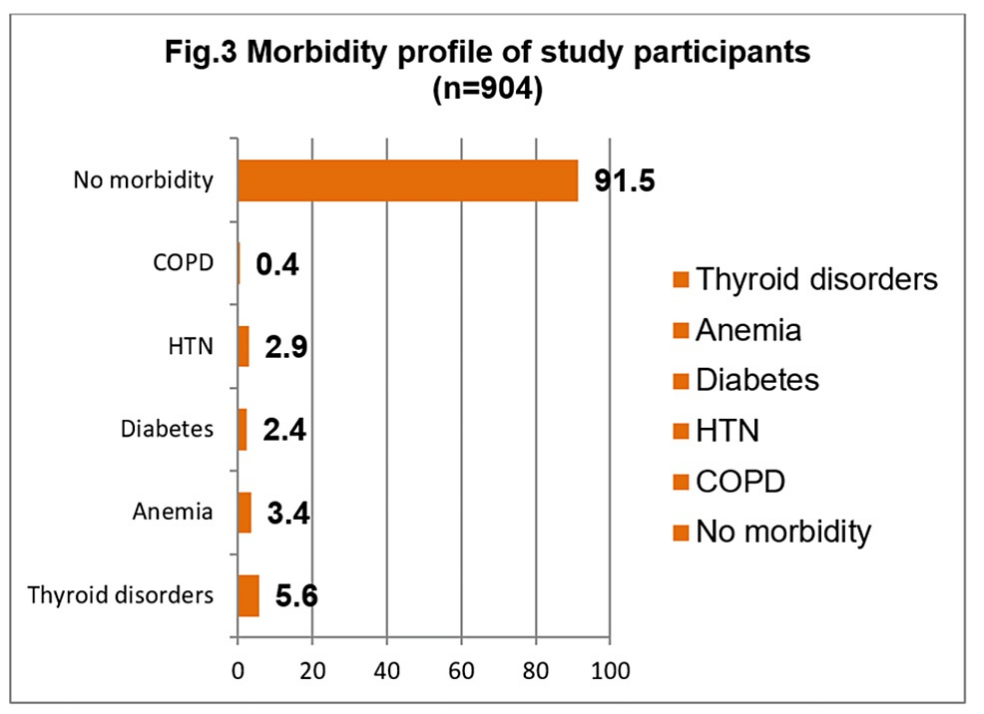
Morbidity profile of study participants (n = 904)

Out of 904 study participants, 6% had a history of COVID-19 infection, and among them, 96% were not vaccinated. About 14% of the study participants had at least one morbidity. Thyroid disorders were the most commonly reported morbidity, followed by anemia, hypertension, and diabetes (Figure 3).

**Figure 4:**
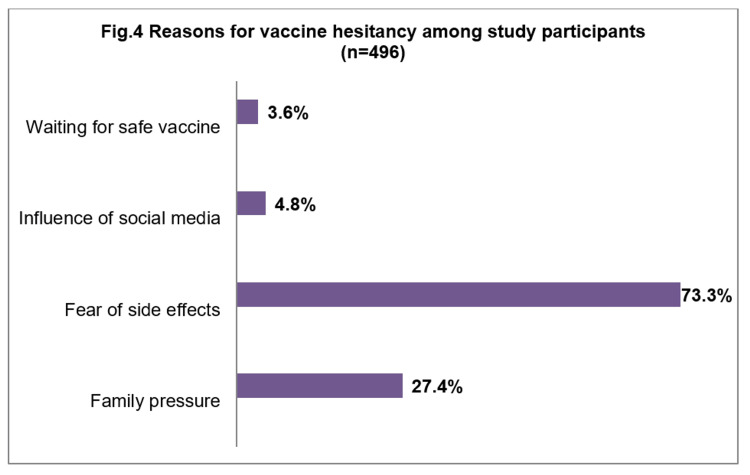
Reasons for vaccine hesitancy among study participants [n = 496]

The common reasons for not accepting the vaccine were “fear of side effects for baby and mother” and “family pressure” (Figure 4).

The most common reasons for accepting vaccines include “good and safe vaccine available”, “motivation of family members and friends,” followed by “motivated by healthcare workers/doctors,” and “availability of vaccine in government health centers at free of cost.”

## Discussion

In the present study, more than 50% of the pregnant and lactating mothers exhibited vaccine hesitancy. Vaccine hesitancy is more common among pregnant and lactating women because of the fear of side effects for the mother as well as the baby. Maternal age > 30 years, primigravidas, breastfeeding mothers with < 6 months old children, lack of awareness, and recent history of COVID-19 infection were substantially associated with poor vaccine uptake. Notably, fear of side effects and family pressure were the most common self-perceived reasons for vaccine hesitancy.

This study showed that the awareness level regarding the eligibility for COVID-19 vaccines among the study participants was 87%, which is higher than the study from North India (68%) [9]. The prevalence of vaccine hesitancy among pregnant and lactating mothers in the present study was 55%. A systematic review and meta-analysis from Italy by Bianchi FP et al. reported that the vaccine hesitation rate among pregnant and breastfeeding women was 48.4% [10]. Studies from the USA and Ethiopia showed that the prevalence of vaccine hesitancy among pregnant and lactating women was 53% and 57%, respectively [2,10]. Among those who received the vaccine, 15.4% were fully vaccinated (two doses), which is slightly less than the study from North India [9].

A systematic review from Italy reported that the worldwide acceptance rate of the COVID-19 vaccine among pregnant women was 50%, which is nearly identical to the present study, but in the case of lactating women, the vaccine acceptance rate was lower in our study compared to the worldwide acceptance rate. The Italian systematic review also reported that 52% of pregnant women were willing to receive the COVID-19 vaccine in their online survey, and the most common reason for vaccine hesitancy was ‘unwanted side effects to the developing fetus,’ which is similar to the present study [11,12].

Various studies reported that COVID-19 vaccine acceptance rates among pregnant and breastfeeding mothers were between 29.7% and 77.4% [13]. This difference in acceptance rates would be due to the different study settings and time of the study. The highest acceptance rate was observed in a study conducted in China, followed by studies in Qatar and Italy [13]. On the other hand, the lowest acceptance rate was found in studies from Switzerland and Ireland [13,14].

A multi-national study reported that nearly half of the study participants had vaccine hesitancy at the end of the first wave, and pregnant women had higher hesitancy. They also reported that the first wave disrupted access to health services and had an adverse effect on pregnancy experiences and breastfeeding support for lactating mothers [14,15].

In our study, there was no association between COVID-19 vaccination and religion, educational status, employment status, or socio-economic class. Whereas a multinational study reported that education level and employment status were associated with vaccine hesitancy among pregnant and lactating women [11], and an Ethiopian study reported that higher maternal education had higher odds of vaccine acceptance [10].

An Ethiopian study reported higher odds of willingness for COVID-19 vaccination among lactating mothers who resided in urban areas and who had received above secondary level education [10]. But the current study was executed in a semi-urban area, and educational status was not significantly associated with vaccine hesitancy or acceptance. This reflects that the better availability of health services and the educational status of mothers in our study area had no influence on decision-making among lactating and pregnant women.

Further, the current study showed that age more than 30 years, primi mothers, lactating mothers with less than six months old children, women with no awareness, and recent COVID-19 infection were independently associated with vaccine hesitancy among study participants. Archanakumari et al. revealed that the first trimester of pregnancy, unforeseen future effects of the vaccine on the unborn baby, and concerns regarding breast feeding were hallmark barriers for COVID-19 vaccination among pregnant and lactating women [9].

In the present study, fear of side effects for the baby and pressure from other family members were the most reported reasons for vaccine hesitancy. Public health experts, stakeholders, and healthcare professionals should address the anxiety and myths associated with vaccination against COVID-19 among pregnant and lactating women. Reliable information from qualified medical personnel on the current state of knowledge regarding safety, effectiveness, and individual-level counseling, along with the recommendations of scientific societies, may contribute to a wider acceptance of vaccination against COVID-19 among pregnant and lactating women. Since social media is one of the most powerful tools to change people’s attitudes, the government should consider using these platforms to deliver authentic information about vaccination among pregnant and lactating women to alleviate vaccine dropout rates and improve vaccine acceptance. Government officials should take the necessary steps to spread credible and reliable information about the vaccine’s development, its efficacy and safety, the time needed to provide protection, and the significance of herd immunity. In addition, collaborations with local community leaders and/or celebrities can be done to influence general and high-risk populations’ decisions on getting vaccinated.

Despite the best efforts by the government and health sector to increase public awareness and acceptability of COVID-19 vaccines, the rate of vaccine uptake in pregnant and lactating women is still low. It was also emphasized that misinformation from unreliable sources plays a major role in vaccine hesitancy in all age groups, including pregnant and lactating women [16,17].

To the best of our knowledge, this was one of the very few studies across India that made an honest attempt to study COVID-19 vaccine hesitancy among pregnant and lactating women. It involved pregnant and lactating women attending government PHCs, CHCs, and GHs in Karaikal district. As we had covered more than 80% of the government healthcare centers in Karaikal, the study findings would be generalizable to the entire Karaikal district and Puducherry, UT. The identified barriers associated with COVID-19 vaccine uptake would benefit the health authorities in analyzing the gaps in vaccine acceptance and uptake. Besides, there is a dire need to build vaccine literacy among the target population. 

As this present study reflected the self-reported vaccine hesitancy and associated factors among pregnant and lactating mothers, in-depth interviews or focus group discussions are needed to understand the sensitive or psycho-social barriers hindering COVID-19 vaccine acceptance. Since it was a cross-sectional study, temporality could not be established. Self-reported adverse events and self-perceived reasons for vaccine hesitancy are subjective and not cross-verified.

## Conclusions

COVID-19 vaccine acceptance and uptake among pregnant and lactating mothers in the present study were not satisfactory to build herd immunity. In addition, higher maternal age, primigradvidas, lactating mothers with less than six-month-old children, ignorance, and a recent history of COVID-19 infection emerged as significant predictors for vaccine hesitancy. Besides, fear of side effects for the unborn child and normative beliefs substantially contributed to the poor vaccine acceptance and uptake. Further, context-specific behavior change communication along with community participation would improve COVID-19 vaccination.
